# EDM of Ti-6Al-4V under Nano-Graphene Mixed Dielectric: A Detailed Investigation on Axial and Radial Dimensional Overcuts

**DOI:** 10.3390/nano12030432

**Published:** 2022-01-27

**Authors:** Kashif Ishfaq, Muhammad Asad, Muhammad Harris, Abdullah Alfaify, Saqib Anwar, Luciano Lamberti, Maria Luminita Scutaru

**Affiliations:** 1 Department of Industrial and Manufacturing Engineering, University of Engineering and Technology, Lahore 548900, Pakistan; 2016im11@student.uet.edu.pk; 2 Industrial and Manufacturing Engineering Department, Rachna College of Engineering and Technology, Gujranwala 52250, Pakistan; m.harris@massey.ac.nz; 3 Industrial Engineering Department, College of Engineering, King Saud University, P.O. Box 800, Riyadh 11421, Saudi Arabia; aalfaify@ksu.edu.sa (A.A.); sanwar@ksu.edu.sa (S.A.); 4 Dipartimento di Meccanica, Matematica e Management, Politecnico di Bari, 70125 Bari, Italy; luciano.lamberti@poliba.it; 5 Department of Mechanical Engineering, Transilvania University of Brasov, B-dul Eroilor No 29, 500036 Brasov, Romania

**Keywords:** electric discharge machining, Ti-6Al-4V, graphene, nano-particles, radial overcut, axial overcut

## Abstract

Ti-6Al-4V is considered a challenging material in terms of accurate machining. Therefore, electric discharge machining (EDM) is commonly engaged, but its low cutting rate depreciates its use. This issue is resolved if graphene nanoparticles are mixed in the dielectric. However, the control over the sparking phenomenon reduces because of the dispersion of graphene particles. Subsequently, the machined profile’s geometric accuracy is compromised. Furthermore, the presence of nanographene induces different sparks along axial and radial cutting orientations. This aspect has not been comprehensively examined yet and dedicatedly targeted in this study to improve the quality of EDM process for Ti-6Al-4V. A total of 18 experiments were conducted under Taguchi’s L18 design considering six parameters namely, electrode type, polarity, flushing time, spark voltage, pulse time ratio, and discharge current. The aluminum electrode proved to be the best choice to reduce the errors in both the cutting orientations. Despite the other parametric settings, negative tool polarity yields lower values of axial (A_DE_) and radial errors (R_DE_). The developed optimal settings ensure 4.4- and 6.3-times reduction in R_DE_ and A_DE_, respectively. In comparison to kerosene, graphene-based dielectric yields 10.2% and 19.4% reduction in R_DE_ and A_DE_, respectively.

## 1. Introduction

Graphene is one of the best nano-materials in terms of good mechanical strength, high thermal conductivity, transparency, chemical inertness, and high hydrophobicity [1,2]. Due to the aforementioned properties and their multi-functionality, graphene nano-particles are widely employed in a range of applications from electronics gadgets to sensors, supercapacitors, batteries, as well as in medicine for dental and biomedical implants [3,4]. Graphene exhibits 2D-hexagonal array configuration, made from carbon atoms by Sp^2^ hybridization [5]. It is worthy to mention that nano-graphene has admirably captured the research focus in EDM, owing to its excellent electrical characteristics which makes the achievement of a high material rate possible in EDM. However, its addition also creates certain challenges in terms of dimensional overcuts, as the dispersion of these particles widen the plasma channel during the electroerrosion process, which eventually translates in compromised dimensional accuracy. This aspect has not been explicitly studied so far in regard to the nanographene mixed dielectric, which is the core focus in this work.

The production of complex parts for various applications in the biomedical, defense, and aerospace sectors is an essential requirement nowadays. There are numerous requirements that finished parts must fulfill to achieve accurate functionality. Out of all the provisions, the dimensional accuracy of the finished product has great importance. Therefore, it is a primary issue for the industries to produce parts with a high level of precision and reliability [6]. The need for high precision parts further upsurges if the manufactured part is a candidate for critical applications such as in nuclear reactors, aerospace, dies/molds, and biomedical [7]. The requirements of dimensionally precise components has increased due to the demand for assembly work in the aforesaid application areas [8]. Advances in manufacturing technology have demanded approaching new materials possessing remarkable mechanical and physical properties. To meet this challenge, titanium and its different alloys, particularly Ti-6Al-4V, have appeared as an appealing choice because of their higher strength to wear ratio, corrosion resistance, and strength retention even at elevated temperature [9,10,11,12]. These novel characteristics make these materials a correct selection for strategic industrial applications such as bio-medical, automotive, and aerospace [13,14]. Regardless of the previously mentioned benefits, the machinability of titanium alloy (Ti-6Al-4V) with high geometrical accuracy is however troublesome if traditional techniques are employed. Ti-6Al-4V is classified as hard-to-cut material due to its high chemical reactivity, and low thermal and chemical conductivity [15]. Yamane et al. [16] reported that low heat conductivity of titanium alloys raised the heat at the tool interface, thus lowering the strength of the cutting edge and reducing the tool life. Temperature increasing at the tool interface causes titanium alloys to stick with the workpiece surface and generate a volatile accumulated edge that eventually deteriorates the machined surface [17]. Hence, machining of Ti-alloys with ultra-precise dimensional accuracy via conventional cutting techniques is very often unaffordable [18]. Thereof non-conventional machining processes are engaged for cutting of Ti-6Al-4V. Amongst non-traditional methods, electric discharge machining (EDM) is promisingly considered because of its high dimensional accuracy and its capability to machine complex shapes without burrs in the work surface of any hardness [9,19].

The EDM process, also nominated as an electro-thermal machining process, is one of the most frequently used non-conventional techniques to cut hard materials such as Ti-6Al-4V [20]. The EDM process generates heat due to repetitive electrical sparking that occurs in the localized region. The intense localized heat melts and vaporizes the workpiece material. The debris thus formed is flushed away by the dielectric medium [21]. Its inherent attribute of utilizing thermal energy to machine the conductive parts without considering hardness has now become its unique advantage for fabricating dies and molds, aerospace, nuclear, automobiles, and surgical components [22]. Moreover, there is no direct contact between the workpiece surface and tool, which reduces the occurrence of oscillations, engineering stresses and chatter during machining [23,24]. Therefore, titanium and its different alloys, especially Ti-6Al-4V, can be effectively machined with the help of electric discharge machining [25,26].

Although EDM offers a variety of benefits, its use is curtailed owing to its low material removal rate (MRR). This issue leads researchers to consider the option of powder additives to achieve better cutting rate [27]. Unfortunately, the mixing of powder particles amplifies the inherent issue of overcut that affects the accuracy of the machined part. Furthermore, it was claimed that the attained dimensions (axial and radial) of a machined surface are somehow larger than the actual diameter of electrode, which induced errors in the cutting orientations [18,28]. Researchers have investigated the issue of overcut during EDM of different materials [29,30,31]. For instance, Muthukumar et al. [32] developed a model for radial overcut (R_OC_) using the response surfaces method (RSM) during machining of Inconel 800. In the same way, Bhaumik et al. [33] developed a semi-empirical model for R_OC_ and found a correlation between numerical and experimental results. It was also claimed that dimensional accuracy has a significant impact on product aesthetics. In another work, the influence of the tool rotation on the magnitude of overcut was investigated. It was reported that high tool rotation affects the overcut value [34]. Kumar et al. [35] indicated that discharge current, spark voltage, and pulse off time have a significant impact on overcut (OC) in EDM of EN19 workpiece. Multiple studies were carried out to analyze the effect of different EDM parameters, including discharge current, spark voltage, pulse-on time, pulse-off time, flushing time, electrode types, and polarity on the OC [36,37,38,39]. However, the significant factors which noticeably affect the magnitude of OC are discharge current and pulse-on time [38,39]. Researchers have reported that the size of OC is reduced by increasing discharge current and pulse-on time due to the high amount of spark energy affiliated with them [40,41]. Contrarily, a reverse trend (i.e., low intensity of discharge current yielded poor dimensional accuracy) was noted between OC and discharge current during EDM of Ti-6Al-4V [42]. Prasanna et al. [43] assessed the impact of different input parameters, including peak current, duty factor, and pulse-on time on OC and tool wear rate (TWR) during EDM of Ti-6Al-4V using copper electrode coated with Al_2_O_3_-TiO_2_. They concluded that peak current is the principal factor controlling the TWR and OC. Furthermore, it was claimed that the proposed parametric setting provided 92% and 62.5% reduction in TWR and OC, respectively. Another investigation inferred that tool material and pulse on time are the most critical parameters for deciding the OC magnitude [44]. In another research conducted on EDM of Ti-6Al-4V, analysis of variance and grey relational analysis was performed. It was concluded that spark voltage is the significant input parameter which affects OC value [45]. The role of cryogenic treatment was also examined in the context of OC during EDM. It was stated that cryo-treated electrode enhances the radial overcut value. Another experimental study analyzed the effect of mixing aluminum, graphite, and silicone particles in the dielectric on the EDM of Inconel 625 done with copper electrode. It was demonstrated that R_OC_ was minimized in case of silicone powder mixed dielectric because of its high electrical resistivity (6.4 × 10^2^ Ω/m) followed by aluminum (2.8 × 10^8^ Ω/m) and graphite (1 × 10^5^ Ω/m) [46]. Batish et al. [47] studied the effect of using different concentrations (0 g/L, 5 g/L, and 10 g/L) of powder mixed dielectric on OC during machining of AISI 1045 steel; three different electrodes (i.e., graphite, brass, and tungsten copper) were considered. They concluded that the size of OC increases for higher powder concentration as it causes an intense discharging between the working tool and workpiece.

A careful review of the literature reveals that the issue of overcut has not been comprehensively investigated so far for the EDM of Ti-6Al-4V, especially when nano-graphene is employed in the dielectric. Addition of nano-graphene into dielectric allows an increase of the cutting rate by a great extent. Therefore, authors have already discussed the role of graphene nano-particles during evaluation of material removal rate and tool wear rate by EDM process as long as Ti-6Al-4V is used as workpiece material [48]. However, based on the previous findings it has also been noted that the inclusion of such additives reduces the control over the spark discharges. The poor control of the sparking phenomenon translates into inferior geometrical accuracy of the machined profile. Moreover, it also causes dissimilar overcut magnitudes in different cutting orientations. This aspect was not assessed in the previous study [48] and thereof authors extend their work to comprehensively investigate the role/influence of graphene addition on the dimensional accuracy of the machined cavity. It should be mentioned that overcut magnitude is of paramount importance in governing the dimensional accuracy of the cut cavity. Especially, in the case of EDM of Ti-6Al-4V, which has critical applications, the aspect becomes more important. Therefore, this study analyzed the effect of six EDM parameters on the values of overcut in radial and axial orientations to ensure the geometrical accuracy of the machined part.

Three electrode materials were used to identify the best electrode choice for the accuracy perspective. Experimentation was performed under Taguchi’s L18 design using nano-graphene mixed into the kerosene dielectric. The results of the experimentation are discussed thoroughly in this article with the help of physical microscopic evidence. Finally, a parametric combination has also been proposed that warrants the minimum radial and axial dimensional errors in Ti-6Al-4V during the EDM with graphene mixed dielectric.

## 2. Materials and Methods

Ti-6Al-4V is taken as benchmark material because of the variety of applications in the automotive, chemical, biomedical, and aerospace industries [11]. In this study, composition of Ti-6Al-4V was confirmed through optical emission spectrometry (see Table 1). Nominal properties of Ti-6Al-4V reported in the literature are tabulated in Table 2 [15]. The dimensions of the workpiece used in this research are 90 mm length, 80 mm breadth, and 15 mm thickness. The machining of Ti-6Al-4V has been performed by three different electrodes (each of 9 mm diameter) made of aluminum (Al), brass (Br), and copper (Cu).

**Figure 1:**
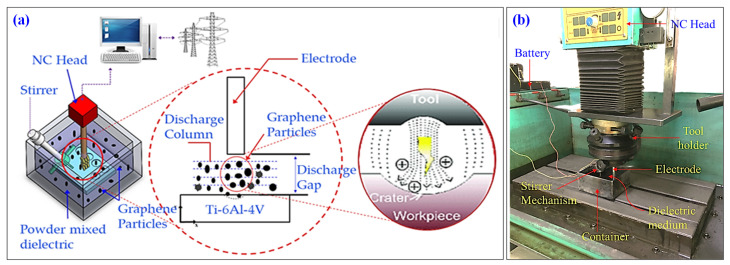
EDM setup: (**a**) schematic of EDM process under graphene mixed dielectric; (**b**) actual machining environment.

Die-sinking EDM equipment (model: RJ-230 made by Creator, Taiwan), as shown in Figure 1b, has been utilized to conduct the experiments. A separate container has been designed to incorporate the graphene nanoparticles in the kerosene dielectric. The schematic of the experimental setup for the machining of Ti-6Al-4V is shown in Figure 1a. In this experimental arrangement, a stirrer was used for mixing the graphene powder in the kerosene oil; the stirrer ensured thorough mixing of the nanoparticles in the dielectric medium. Micro-impressions of depth 0.2 mm were machined using electrodes of 9 mm diameter. The presence of nano-graphene in the dielectric determines the resultant cutting rate, but also limits the control over the sparking phenomenon. The dispersion of the sparking activity may lead to compromise geometrical accuracy of the machined cavity.

In this work, the machinability of titanium alloy was examined in terms of geometric accuracy by considering radial dimensional error (R_DE_) and axial dimensional error (A_DE_). Six input parameters, i.e., tool polarity (TP), type of electrode (E), spark voltage (SV), discharge current (DC), pulse time ratio (PTR), and flushing time (FT) have been selected herein to comprehensively investigate the Ti-6Al-4V machinability issue. The selection of these input parameters was based on two criteria, i.e., either the impact of a particular variable on the defined responses is yet not assessed, or it has well-proven effect with respect to the output variables. For instance, DC, PTR and SV significantly affect the defined responses [49]. Whereas, the impact of TP, E and FT has not been thoroughly investigated yet for the set responses and hence are considered here for investigation. The rest of control factors (servo-sensitivity, spark time, and powder concentration) were set as constant parameters in this research.

Preliminary trials were performed before the actual experimentation for assessing the effect of input parameters. These trials served to set proper values of input parameters so to obtain complete machined impressions. Furthermore, it was also focused that the burn marks of excessive burning from a tool or workpiece should not occur for the chosen ranges of variation of input parameters. Hence, only parameter settings yielding a minimal chance of burn marks and incomplete machining impression were considered in the final experimentation.

The defined levels for each of the six input parameters used in the final experimentation are tabulated in Table 3. Experiments were carried out according to the Taguchi L18 orthogonal design of experiments, one of the best methods reported in the literature [50]. For the selected levels of control variables, a total of 18 experiments were performed with the graphene-based dielectric. Parameter combinations resemble those of Reference [48] and hence are not reported here for the sake of brevity. The powder of graphene nanoparticles was added in a concentration of 0.5 g/L into the dielectric kerosene. The choice of graphene nanoparticles was motivated by their outstanding mechanical, chemical and electrical properties [51,52,53]. The relevant properties of graphene nanoparticles are listed in Table 4.

**Figure 2:**
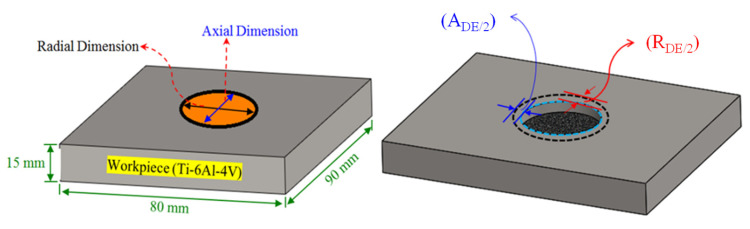
Schematic of radial and axial dimensions and definition of the corresponding errors.

**Figure 3:**
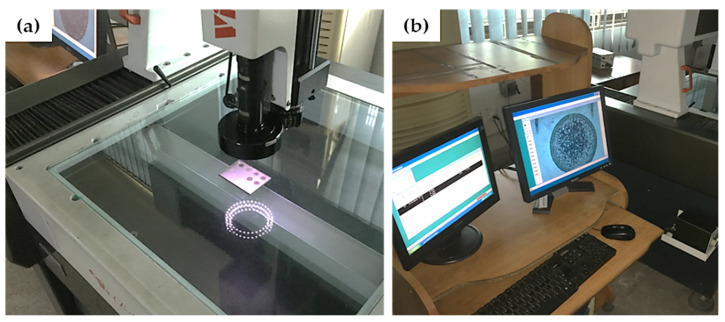
Coordinate measuring machine: (**a**) workpiece setting on CMM, (**b**) dimensional measurement of machined surfaces.

It should be mentioned that pulse on time and pulse off time were treated individually in the previous investigations [48]. However, in this research, pulse time ratio (PTR), which is defined as the ratio of pulse-ontime to pulse-off time, has been selected as an input parameter. Experimentation was done as per the L18 design in a randomized manner. Three replications were performed for each experiment corresponding to a selected combination of input parameters in order to have statistically significant results. After completion of every trial, the diameters of the machined impression were measured along radial and axial directions by a coordinate measuring machine (CMM). The diametric values of machined impressions were used to calculate the machining errors in radial and axial directions. The radial dimension error (R_DE_) and axial dimension error (A_DE_) are simply defined as the difference between diametric values along the radial/axial directions of the machined impression and the diameter of the electrode. The schematic of R_DE_ and A_DE_ is presented in Figure 2. The radial and axial diametric values were measured using a CMM (see Figure 3). Errors R_DE_ and A_DE_ were determined as:

Diametrical Errors = MD (mm) − AD (mm)(1)

where MD and AD are the measured diameter of the machined cavity and the actual diameter of the tool, respectively.

After calculating the magnitudes of R_DE_ and A_DE_, the results have been broadly investigated using a statistical approach based on parametric plots. The values of R_DE_ and A_DE_ were noted against all parametric levels of the selected control variables. Since polarity may have two states (i.e., positive or negative polarity), nine experiments were conducted at positive polarity and the other nine at negative polarity.

Similarly, the electrode type has three levels; hence, six experiments were performed with each electrode (aluminium, brass, and copper). Based on this analogy, the rest of EDM input parameters were examined. From the analysis point of view, the mean values of each parametric level were calculated. A comprehensive discussion was made on the basis of the evidences given by optical microscopy for a detailed explanation of the findings in EDM of Ti-6Al-4V using graphene-based dielectric. Finally, an optimal setting of process parameters was obtained via the grey relational approach (GRA) for minimizing the errors’ magnitude in both cutting orientations. The details of the GRA approach are given in the forthcoming sections.

**Figure 4:**
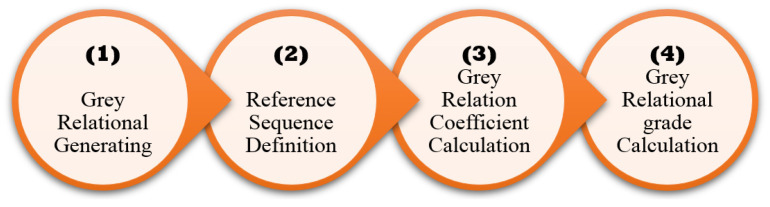
Procedure for GRA.

GRA is a multi-objective analysis that has been proven suitable and user-interactive for solving complex problems involving correlation between various control variables and output responses. This technique consists of various steps, as demonstrated in Figure 4.

In the very initial phase (grey relational (GR) generating), all the performance alternatives have been turned into comparability sequence by scaling them from 0 to 1. Three relationships are established: smaller the better, larger the better, and concentrate on the better. The results of the selected outcome response are evaluated based on narrated relationships. For example, in the current study, the R_DE_ and A_DE_ have been evaluated against input parameters. Thus, according to the relationship, the smaller value of both is rated as best. Thereof, the smaller the best criterion is picked as depicted in Equation (2):

(2) $$X_{\mathrm{ij}}=\frac{\mathrm{Max}\;\left\{Y_{\mathrm{ij}},\;i=1,\;2,\ldots ,\;m\right\}-{\;Y}_{\mathrm{ij}}}{\mathrm{Max}\;\left\{Y_{\mathrm{ij}},\;i=1,\;2,\ldots ,\;m\right\}-\;\mathrm{Min}\;\left\{Y_{\mathrm{ij}},\;i=1,\;2,\ldots ,\;m\right\}}.\;$$

For, i=1,2,…m & j=1,2,…,n

where m = alternatives, n = attributes, Y_ij_ = performance of j-th attribute in the ith alternative—denoted by Yi = (Y_i1_, Y_i2_,….., Y_ij_….Y_in_), and X_i_ = comparability sequence.

In the second phase (reference sequence definition), the best alternative is chosen by finding a performance value that is the nearest as possible or equal to 1. In this research, reference sequence X_o_ (X_o1_, X_o2_, ….., X_oj_,…..X_on_) has been taken as (1, 1,….., 1….1). After that, the comparability sequence is calculated by comparing it with the reference sequence.

In the third phase, GR coefficient is calculated using Equation (3), which tells us how much X_oj_ and X_ij_ are akin to each other.

(3) $$\gamma \;\left(X_{\mathrm{oi}},{\;X}_{\mathrm{ij}}\right)=\frac{\Delta_{\min}+\zeta \;\Delta_{\max}}{\Delta_{\mathrm{ij}}+\zeta \;\Delta_{\max}}.\;$$

For i=1,2,…., m j=1, 2, ……,n

where, γ  = GR coefficient, ζ = distinguishing coefficient (its value ranges from 0 to 1), $\Delta_{\min}$ and $\Delta_{\max}=$ minimum and maximum differences between comparability sequence and the reference sequence. $\Delta_{\mathrm{ij}}$ is calculated from Equation (4) while $\Delta_{\min}$ and $\Delta_{\max}$. are computed from Equations (5) and (6), respectively.

(4) $$\Delta_{\mathrm{ij}}=\left|X_{\mathrm{oi}},{\;X}_{\mathrm{ij}}\right|$$

(5) $$\Delta_{\min}=\mathrm{Min}\;\left\{\Delta_{\mathrm{ij}},\;i=1,2,\ldots \ldots ,m;j=1,2,\ldots \ldots ,n\right\}.\;$$

(6) $$\Delta_{\max}=\mathrm{Max}\;\left\{\Delta_{\mathrm{ij}},\;i=1,2,\ldots \ldots ,m;j=1,2,\ldots \ldots ,n\right\}.\;$$

In the last step, grade Γ for GR is enumerated from Equation (7). The GR grade assesses the relationship between comparability sequence and reference sequence value. Thus, a high value of GR grade demonstrates that comparability sequence value is very close to reference sequence. For the given Equation (7), $w_{j}$ and γ denote weight for attribute j and GR coefficient, respectively.

(7) $$\Gamma \left(X_{o},{\;X}_{i}=\overset{n}{\underset{j=1}{\sum }}w_{j}\gamma \left(x_{\mathrm{oi}},{\;x}_{\mathrm{ij}}\right)\right)$$

For i = 1,2,……., m

## 3. Results and Discussion

This section presents the experimental results and their analysis, followed by a comprehensive discussion to investigate the influence of graphene nanoparticles mixed dielectric on the diametrical overcut. The variations of radial dimensional error (R_DE_) and axial dimensional error (A_DE_) are evaluated against the selected EDM input parameters. After obtaining the results, parametric plots were drawn to envisage the trend of the control variable for the set responses (R_DE_ and A_DE_).

**Figure 5:**
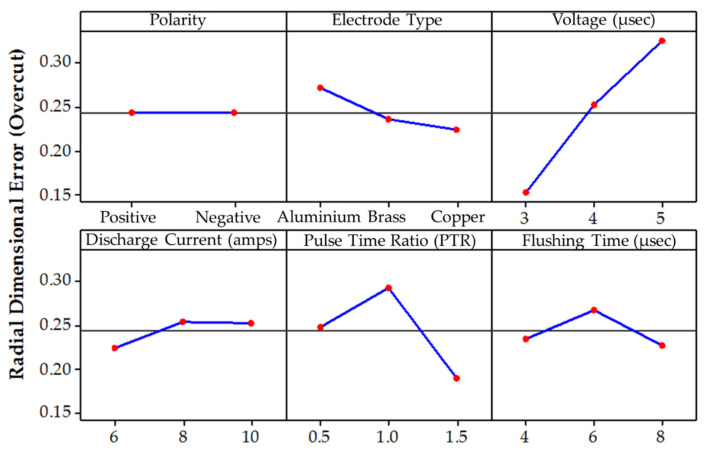
Average experimental results obtained for the radial dimensional error by varying each control parameter for the EDM process including graphene-based dielectric fluid.

**Figure 6:**
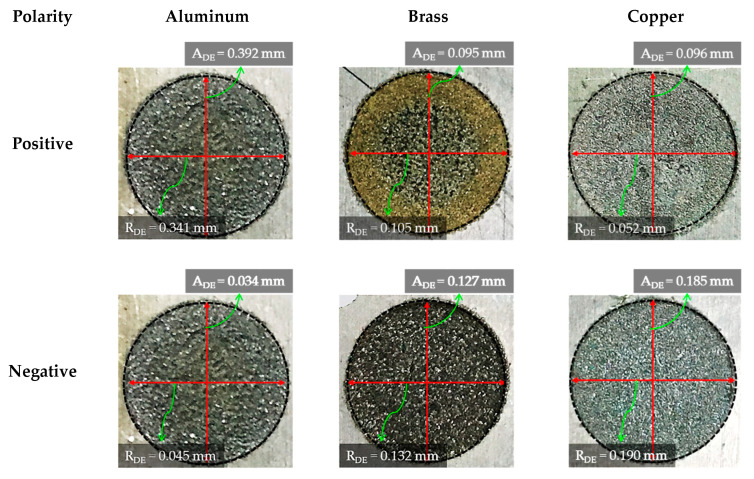
Effect of polarity on minimum values of dimensional errors obtained for each electrode.

**Figure 7:**
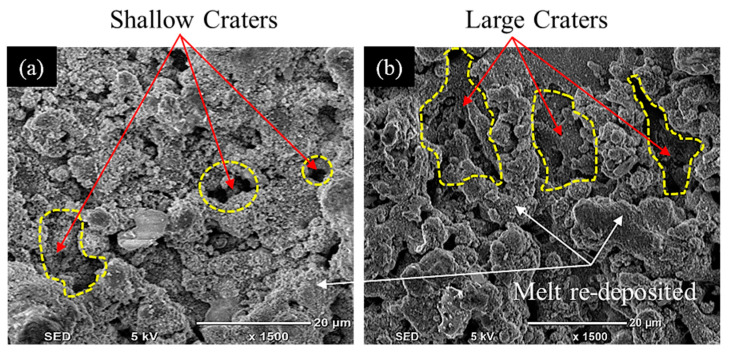
Micrographs representing surface morphology of Ti-6Al-4V specimens machined with Al-electrode at two different tool polarities: (**a**) positive polarity; (**b**) negative polarity.

The die sinking EDM consists of positive and negative polarities. If the workpiece carries a positive charge, then certainly the tool has the negative terminal and vice versa. The effect of varying tool polarity on R_DE_ response during EDM of Ti-6Al-4V is illustrated in Figure 5. It can be seen that the tool polarity slightly affects the mean value of R_DE_ when graphene mixed dielectric is used for the cutting of Ti-6Al-4V. Negative tool polarity allows on average to achieve smaller values of R_DE_ than in the case of positive polarity. The comparison between positive and negative polarity against selected electrodes is also displayed in Figure 6. For instance, aluminium electrode with negative polarity provided the minimal magnitudes of 0.045 and 0.034 mm for R_DE_ and A_DE_, respectively. Contrarily, large values for both errors (i.e., R_DE_ = 0.341 mm and A_DE_ = 0.392 mm) were found for positive polarity, as far as aluminium electrode is concerned. It is mentioned in the literature that less energy is generated between the tool and workpiece gap at negative polarity. Thus, a lower amount of material can be eroded from the central region of the workpiece as well as from the cutting edges of machined cavity. This generated less deep craters on the specimen surface (see Figure 7b); such a result is consistent with the literature [18]. Conversely, for positive polarity, the nano-particles present in the dielectric liquid intensify spark energy and the intense heat generated disintegrates the material thus creating deep craters in the workpiece’s surface (see Figure 7a). This leads to increase R_DE_ error.

**Figure 8:**
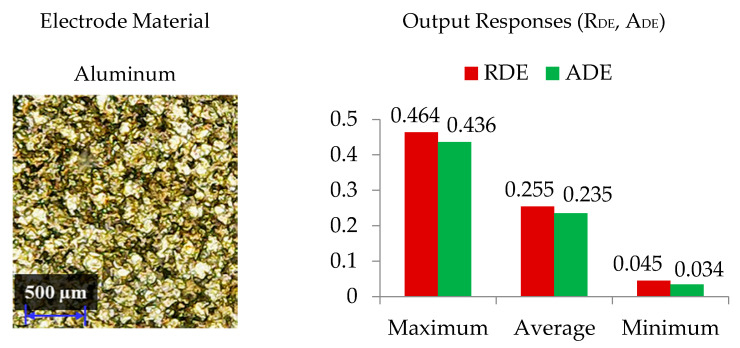

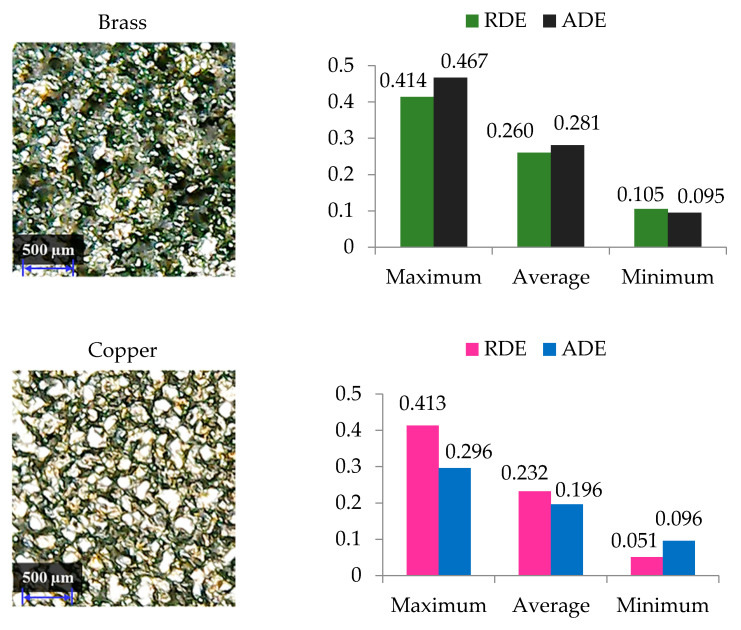
Response magnitudes of electrode types.

**Figure 9:**
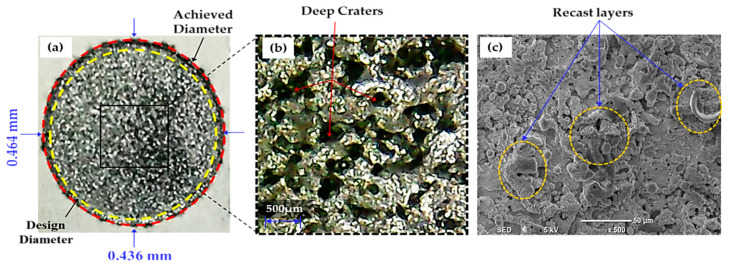
Experimental results for Al-electrode at positive polarity: (**a**) actual machined surface representing a high value of overcut; (**b**) deep craters are present on the workpiece surface; (**c**) SEM image shows re-cast layers formed on the specimen’s surface.

The selection of tool material is also an essential consideration in evaluating R_DE_. Three distinct electrodes (aluminum, brass and copper) were employed in this study, as mentioned previously. The effect of each electrode on the R_DE_ is shown in Figure 5. Moreover, the comparison of selected electrodes against each response’s magnitude is provided in Figure 8. The decreasing trend is perceived for R_DE_ from aluminium to copper electrode. This is because of the higher thermal conductivity of copper (385 W/mK) with respect to brass (109 W/mK) and aluminum (205 W/mK) electrodes. The greater magnitude of R_DE_ seen for the Al electrode is attributed to its lower thermal conductivity which resists the dissipation of heat energy in the tool surface. Thereof the heat stays in the cutting regime and causes the severe melting and vaporization of the workpiece. This effect results in the generation of deep craters with re-cast layer on the workpiece. Since more melting and vaporization of the workpiece material occurs, this tends to increase the R_DE_ as highlighted in Figure 9 [54].

In the case of the copper electrode, the lower value of R_DE_ was obtained. This happens because the graphene nanoparticles included in the dielectric pose a hindrance in front of the spark. Consequently, carbon particles are released in the pool due to the interaction of the plasma with nano-graphene. These particles stick to the electrode surface and hamper the spark strength: hence, the R_DE_ is reduced. Thus, the electrode of Cu would be a preferred choice to have a lower magnitude of R_DE_ in EDM of Ti-6Al-4V.

**Figure 10:**
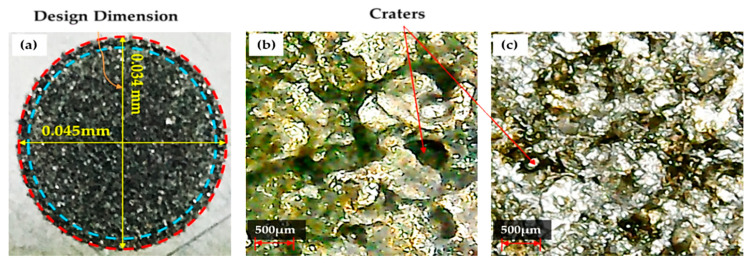
Experimental outcomes for Al-electrode with 3 V spark voltage: (**a**) actual machined surface characterized by R_DE_ = 0.045 mm and A_DE_ = 0.034 mm; (**b**) micrograph showing the smaller number of craters present on workpiece surface; (**c**) micrograph of the electrode surface showing the presence of only one or two craters.

Spark voltage (SV) is the most significant input parameter considered in this study affecting the dimensional accuracy of the worked part. Its effect on R_DE_ is presented in Figure 5. It can be seen that R_DE_ increases almost linearly with the magnitude of SV. Therefore, the smallest level (3 V) of SV is considered as the most reliable choice for getting high geometric precision of the manufactured parts. In fact, at low voltage, limited discharging occurs in the gap between workpiece and tool, thus reducing the material erosion rate [32]. Consequently, the size of R_DE_ is reduced as it appears from Figure 10. Whereas, at a large value (i.e., 5 V), the nano-particles present in the dielectric liquid increased the current flow in the machining region. The higher current amplitude enlarged the effective width of plasma channel thus generating a larger amount of discharge energy in the cutting zone. The plasma channel was dispersed beyond the cut dimensions owing to the presence of tiny graphene particles. The resulting overcut yield higher values of R_DE_ and A_DE_.

**Figure 11:**
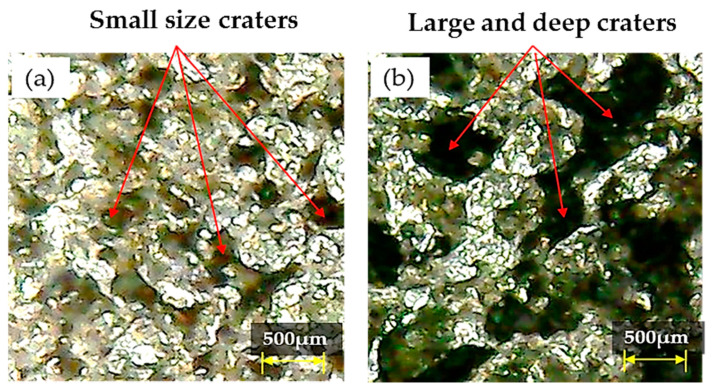
Micrographs showing size of craters in the specimen surface treated with brass electrode: (**a**) Small and deep craters formed at 6A discharge current; (**b**) Large and wide craters formed at 8 A discharge current.

Another important parameter is the discharge current (DC) whose effect on R_DE_ is illustrated by Figure 5. It is understood that, at higher DC values, the presence of graphene nanoparticles in the dielectric enhanced the strength of the spark, which transfers more energy to the machining zone. Hence, melting of the workpiece is more pronounced and this increases the R_DE_. Besides worsening R_DE_, the high temperature established between the electrode and workpiece also caused larger and wider craters to form on the workpiece surface, as displayed in Figure 11 [55]. However, an interesting phenomenon is seen passing from 8 to 10 A, the R_DE_–DC curve becomes approximately horizontal (see Figure 5), which means that there is no further appreciable change in R_DE_. Hence, 6 A is the best DC value for maximizing accuracy of EDM machining of Ti-6Al-4V.

**Figure 12:**
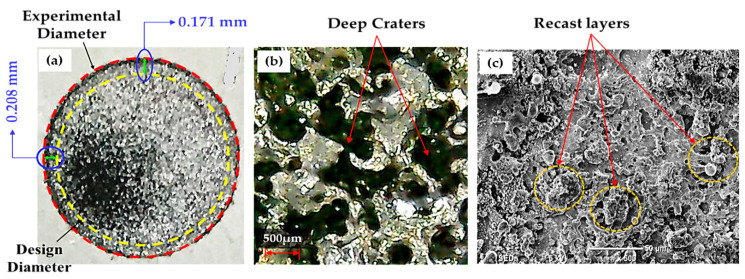
Experimental results obtained for the Cu-electrode and PTR = 1.5: (**a**) machined surface of workpiece; (**b**) micrograph of Ti-6Al-4V showing the presence of deep craters on the workpiece surface; (**c**) recast layers occurred on tool surface.

The variation of R_DE_ with respect to pulse time ratio (PTR) also can be analyzed looking at Figure 5. In this research, the value of pulse-off time (50 µs) remained constant for all experiments. The R_DE_ increased as PTR passed from 0.5 to 1.0 and then decreased sharply when PTR passed from 1 to 1.5. The initial increase of R_DE_ was related with the spark energy generated in the cutting regime [55]. A greater magnitude of spark energy is realized for PTR = 1 as the pulse-ontime increased from 25 to 50 µs. This liberated more heat in the spark gap that increased the melting of material. However, increasing further the pulse-ontime to 75 µs, R_DE_ was significantly reduced due to the discharge of graphene particles at the higher energy peak. This led to the deposition of the particles’ layer onto the tool surface. This layer acts as a shield over the tool periphery and hence the sparking efficacy of the electrode is compromised. Subsequently, a lesser amount of material is eroded and this allows a reduction of the R_DE_. Furthermore, high PTR values also ensure the existence of the melt pool for a longer period, which minimizes the probability of debris re-deposition. However, too large values of PTR (i.e., 1.5) cause the formation of deep craters on the workpiece surface (see Figure 12).

The parametric plots of Figure 5 demonstrate that the effect of flushing time (FT) is less significant in comparison to DC and SV. It can be seen that the lowest R_DE_ value is obtained for the very large value of FT. Interestingly, the 1st level of FT (i.e., 4 µs) also allowed to obtain a very small R_DE_ value but yet larger than that achieved for FT = 8 µs. The highest value of R_DE_ was obtained for FT = 6 µs. Such a value probably allowed to efficiently remove debris without a significant quenching. Hence, a larger amount of material was removed from the target surface, thus increasing R_DE_. However, for FT = 8 µs, the graphene particles present in the dielectric were deposited on the tool surface because the tool and workpiece are submerged in the dielectric. As FT becomes longer, the probability of re-deposition on the machined area increases as quenching might occur both in the tool and workpiece. This reduces the spark intensity concentration and the material erosion rate, thus leading to have a lower value of R_DE_.

The best setting of parameters yielding the minimum values of R_DE_ are: polarity = negative, tool material = copper, SV = 3 V, DC = 6 A, PTR = 1.5, and FT = 8 µs.

**Figure 13:**
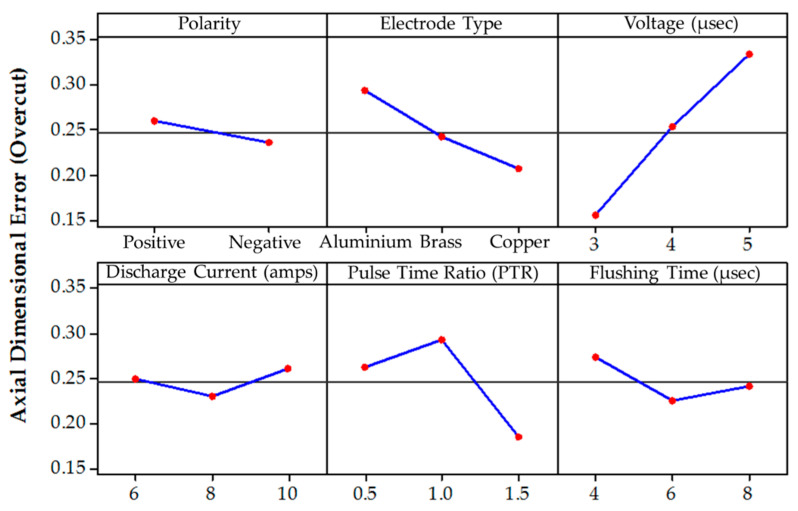
Average experimental results obtained for the axial dimensional error by varying each control parameter for the EDM process including graphene-based dielectric fluid.

The effects of all the aforementioned control variables on A_DE_ are described by Figure 13 for the EDM set up including the graphene particles mixed dielectric. As mentioned earlier, two types of polarity (i.e., positive and negative) were considered. The averaged experimental results obtained for different polarities indicate that the value of A_DE_ decreases when polarity turns from positive to negative (see Figure 13). For positive polarity, the energy produced in the dielectric is absorbed by the graphene nanoparticles. This stabilizes the sparking in the spark gap [56] and more material is removed from the workpiece, thus raising the magnitude of A_DE_. Conversely, at negative polarity, less material is removed because the nano-particles present in the dielectric medium cause dispersion of heat in all directions, and thus A_DE_ is reduced.

**Figure 14:**
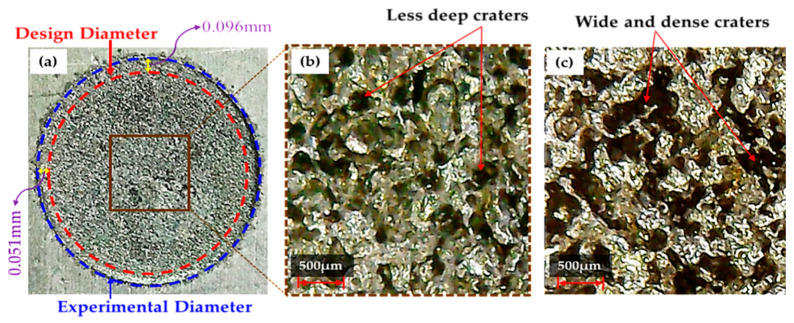
Experimental results obtained for the Cu-tool: (**a**) actual machined surface of workpiece showing minimum value of overcut; (**b**) micrograph showing less craters on the surface of the workpiece; (**c**) surface of the Cu tool representing wide and dense craters.

The effect of electrode material on A_DE_ (see Figure 13) is similar to the observed trend for R_DE_ (see Figure 5). The dimensional error decreased passing from the aluminum electrode to the copper electrode. The Cu electrode is the most efficient one because the carbon particles produced by the discharging stick to the electrode surface and make material removal rate decrease. However, the surface of the Cu tool deteriorated because of the discharging of graphene particles over the surface, which produced more irregularities on it (see Figure 14).

**Figure 15:**
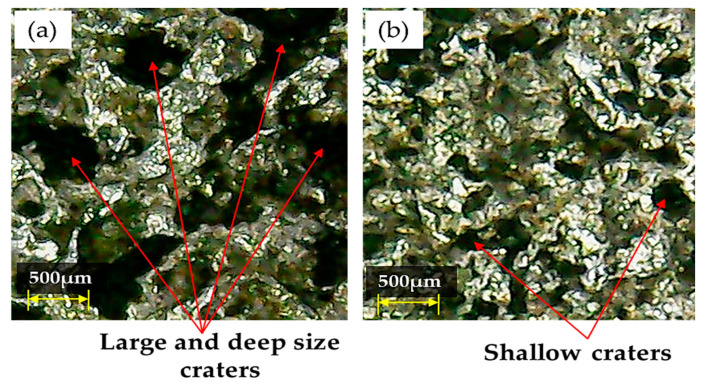
Micrographs demonstrating different types of craters formed on the workpiece surface with: (**a**) Al-electrode; (**b**) Cu-electrode.

Since the Al electrode has a lower melting temperature (660 °C) than Brass (932 °C) and Cu–electrodes (1084 °C) it wears out more rapidly. In EDM, the machined impression is a replica of the tool’s profile. Hence, a worn electrode surface provides poor control over the sparking phenomenon in the EDM of Ti-6Al-4V using nano graphene-based dielectric. This results in larger A_DE_ values. Since sparking occurs in an unstable manner in graphene mixed dielectric that has a capability to uplift the spark potential, the quality of the machined surface is compromised and presents large and deep craters (see Figure 15a). However, Cu-electrode caused the formation of shallow craters (see Figure 15b) due to the adhesion of small carbon and graphene particles onto the tool surface, which lead to stable discharging between the work-electrode gap. Consequently, A_DE_ improved. The experimental results gathered in this study prove that, under the powder mixed dielectric, copper tool is more effective in terms of mean R_DE_ and A_DE_ in the cutting of Ti-6Al-4V.

Interestingly, SV significantly affects the micro-machining errors in both the cutting orientations, as highlighted by Figure 5 and Figure 13. The A_DE_ and R_DE_ errors sharply increase with SV. A similar effect of SV was also noted while investigating the TWR of the EDM setup with nanographene mixed dielectric (see Reference [48]). At the 1st level selected for SV (i.e., 3 V), the rate of ionization for graphene particles is low. Hence, a lower discharge energy is produced which causes plasma density to decrease in the region comprised between workpiece and tool. Due to minimal plasma density in the machining gap, the amount of material removed is smaller. Consequently, the value of A_DE_ is significantly reduced. However, at the 3rd selected level for SV (i.e., 5 V), spark intensity is enhanced in the dielectric medium. Such a rise in the strength of discharge energy led towards a greater pool of ions due to the increase in ionization of graphene nano-particles over the machined surface. These ions strike the surface of workpiece and erode more material; hence, A_DE_ is increased [57]. Thus, 3 V is the optimal level for achieving high surface integrity with the precise dimensions of the workpiece.

**Figure 16:**
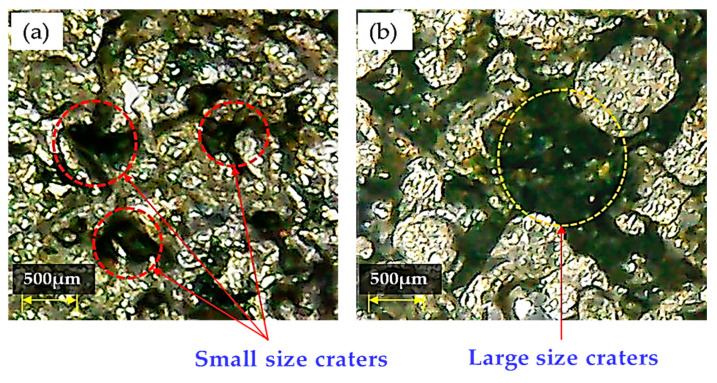
Micrographs showing the different sizes of craters present on workpiece’s surface machined witha brass tool: (**a**) small size craters generated for DC = 8 A and positive polarity; (**b**) large size craters generated for DC = 10 A and negative polarity.

The value of A_DE_ is also sensitive to the variation of DC if Ti-6Al-4V is machined through EDM with graphene mixed dielectric (see Figure 13). Increasing DC from 6 to 8 A allowed A_DE_ to be reduced. This is due to the presence of graphene nano-particles that influence the discharging process by creating hindrance in front of sparks. The hurdle created by particles decreases the discharge energy. This reduced the MRR leaving small size craters on the workpiece surface (see Figure 16a). Hence, the magnitude of A_DE_ drops down as DC increases. However, A_DE_ sharply increased to its maximum value for DC = 10 A due to an intense heat generation in the discharge gap. At 10 A, more material is detached from the workpiece due to a large heat input because of powerful sparking in the machining gap [58]. Hence, the discrete sparking hits more strongly on the surface of the workpiece and creates the large craters shown in Figure 16b. In summary, the presence of large craters caused the sudden increase in A_DE_ value.

The variation in A_DE_ is also evaluated against different values of PTR under graphene-based dielectric. Experimental results are plotted in Figure 13. The 3rd level selected for PTR (1.5) yield the lowest dimensional error A_DE_. The effect of PTR appears to be similar for both types of cutting errors R_DE_ and A_DE_. Therefore, the variations of A_DE_ with respect to PTR can be explained with the previous arguments developed for R_DE_.

**Figure 17:**
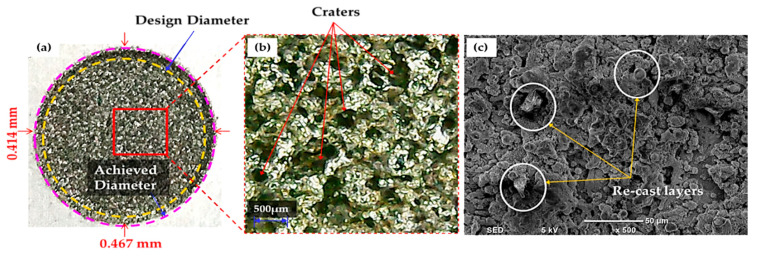
Experimental results obtained for the brass electrode: (**a**) actual machined surface showing a high value of overcut; (**b**) micrograph showing the presence of small size craters formed on the workpiece surface; (**c**) SEM micrograph showing the presence of deep craters on the surface of Ti-6Al-4V.

As the spark on-time raised from 25 to 50 µs to reach the middle level of 1.0 selected for PTR, graphene nanoparticles got discharged and started melting the material more quickly, leaving small size craters on the surface (see Figure 17b) [59]. Hence, A_DE_ increased. On the other hand, increasing further PTR to 1.5 allowed A_DE_ to be reduced because graphene particles burned smoothly and built a re-cast layer on the electrode’s surface (see Figure 17c). This re-cast layer acted as a shield protecting the specimen surface from further damage: consequently, the value of A_DE_ became lower. Therefore, for PTR = 1.5, the EDM process was more uniform, and it was possible to achieve high geometric accuracy.

**Figure 18:**
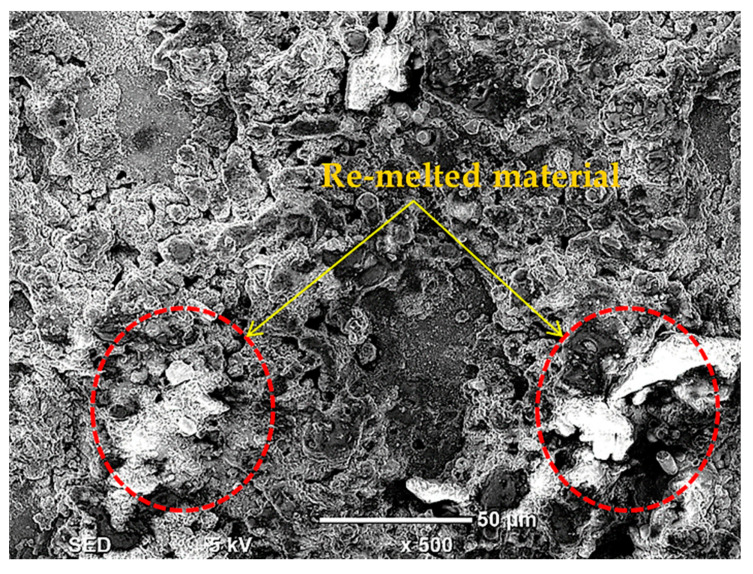
SEM micrograph showing evidence of reposition layers on the workpiece surface.

The variation of A_DE_ with respect to the flushing time parameter FT is shown in Figure 13. A_DE_ dropped down as FT raised from 4 µs to 6 µs but then increased for FT = 8 µs. As mentioned before, longer FT means that more time is provided to the EDM equipment to flush away the debris over the machined surface. The efficient removal of the debris from the machining regime helps to achieve better dimensional control. Hence, the magnitude of A_DE_ could be reduced by increased FT up to 8 µs. However, for FT = 8 µs, the opposite occurred, and A_DE_ increased. Basically, the larger flushing time improved the probability that debris quench on the cut profile. Graphene nanoparticles also contributed to this phenomenon. The re-solidification occurs at the machined cavity in a random manner. Moreover, its effect was more prominent at the cutting periphery of the machined cavity. Therefore, the A_DE_ error increased for the very large value of FT. The re-deposition on the cut surface (machined at FT = 8 µs) is also visible in the SEM micrograph of Figure 18. After having discussed in great detail the effects of EDM process parameters on dimensional errors R_DE_ and A_DE_, the optimal combination of input parameters was developed.

It is worth mentioning that R_DE_ and A_DE_ are different in magnitude and also the influence of some of the parameters is dissimilar for them. Therefore, the grey relational approach (GRA), which is a multi-objective optimization methodology, was used in this research to find the optimal variables’ combination. GRA results are tabulated in Table 5. Based on the findings shown in the said Table 5, the best alternate that can provide lower values of both the errors i.e., A_DE_ and R_DE_ is alternate no. 10. The proposed optimal setting of EDM parameters reported in Table 6 was also tested through confirmatory trials. In order to compare the proposed nano-graphene mixed dielectric EDM setup with the traditional kerosene oil based dielectric setup, the optimal setting yielding the minimum values of R_DE_ and A_DE_ was used also for the traditional kerosene-based set up. Table 7 shows that both errors R_DE_ and A_DE_ achieved by the proposed graphene-mixed EDM set up could be significantly reduced using the optimal setting. In particular, the minimum errors R_DE_ and A_DE_ were respectively 4.4-times and 6.3-times lower than average values while the error difference δ decreased by a factor 4.

**Figure 19:**
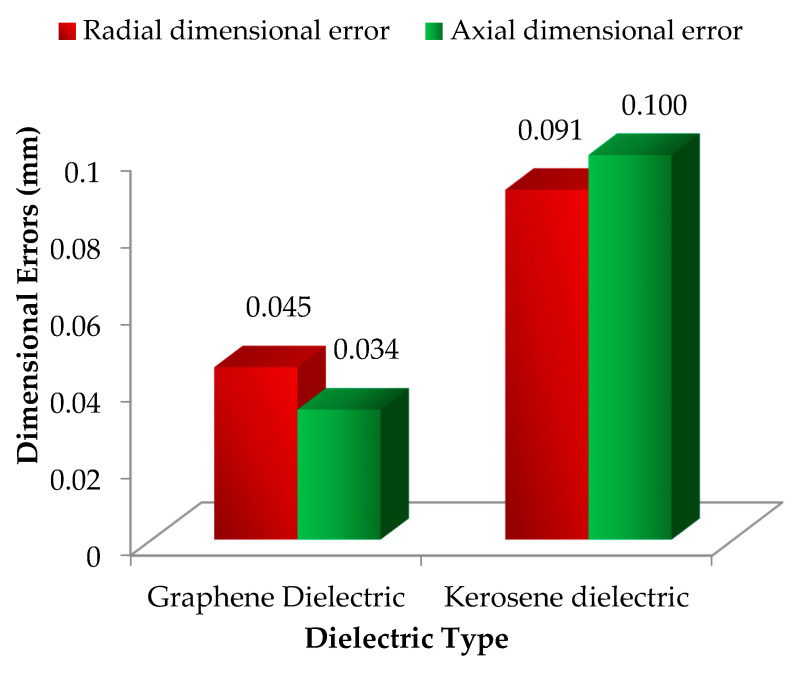
Comparison of minimum EDM’s dimensional errors between graphene and kerosene-based dielectric.

Figure 19 presents the minimum dimensional errors obtained by implementing the optimized EDM’s process parameters of Table 6 for the proposed EDM set up using mixed-graphene and the traditional EDM set up using the conventional kerosene-based dielectric. It can be seen that the conventional EDM of Ti-6Al-4V achieved a poor geometric accuracy resulting in larger dimensional errors in both cutting orientations R_DE_ and A_DE_. In fact, the minimum dimensional errors R_DE_ and A_DE_ achieved by the proposed EDM set up using mixed-graphene were reduced, respectively, by 50% (i.e., only 0.045 vs. 0.091 mm) and 66% (i.e., only 0.034 vs. 0.100 mm) with respect to their counterpart achieved by traditional kerosene-based EDM set up. Furthermore, R_DE_ found with the traditional EDM set up was 10.2% higher than that achieved by the proposed EDM set up at the defined optimal settings mentioned in Table 6. In the same way, A_DE_ was 19.4% larger in magnitude for the traditional EDM set up employing kerosene dielectric. In summary, the proposed mixed-graphene EDM set up achieved significantly higher manufacturing accuracy for Ti-6Al-4V alloy than the classical EDM set up.

## 4. Conclusions

The present research analyzed geometric accuracy issues arising in the EDM of Ti-6Al-4V workpieces machined under graphene-mixed dielectric. The addition of graphene nanoparticles into the dielectric medium improves significantly the cutting rate but reduces the control over the spark discharges. Moreover, the width of plasma channel is also enlarged, inducing dimensional errors. It should be mentioned that dimensional errors for different cutting orientations (i.e., radial and axial) are not of the same magnitude because of varied discharge characteristics in both orientations when graphene-based dielectric is used. These issues play a vital role in governing the dimensional accuracy of the workpiece. However, these problems are not discussed comprehensively in the literature. Therefore, this study deeply investigated the effect of using graphene mixed dielectric on radial (R_DE_) and axial (A_DE_) dimensional errors against six EDM process parameters. Experiments were performed using three electrodes (Al, Brass, Cu) based on Taguchi’s (L18) approach. Experimental results were thoroughly analyzed via statistical tests and microscopy-based inspections. The optimal setting that minimizes dimensional errors in both the cutting orientations with respect to target impression sizes was developed using GRA approach. Based on experimental results, the following conclusions are drawn:

i. The Cu electrode outperforms other electrodes in terms of mean values of R_DE_ and A_DE_ errors.
ii. Amongst the other EDM parameters, spark voltage and pulse-time ratio significantly affect the magnitude of dimensional errors in axial and radial machining orientations. The very small value of spark voltage (i.e., 3 V) helps to restrain the spark discharges in a localized machining region. This allows lowering of the R_DE_ and A_DE_ values down to 0.045 and 0.034 mm, respectively_._ The very large pulse-time ratio (1.5) also allows minimization of machining errors in both cutting directions.
iii. The negative tool polarity allows a reduction of the values of R_DE_ and A_DE_ when the Al electrode is employed in the EDM of Ti-6Al-4V with the graphene-mixed dielectric. However, the reverse occurs if a brass electrode is used.
iv. The desired levels of parameters for minimizing R_DE_ and A_DE_ as well as the difference between errors were developed using GRA approach. The adequacy of the proposed setting i.e., polarity = negative, Tool material = Al, SV = 3V, DC = 6 A, PTR = 0.5, and FT = 4 µs, also was validated by carrying out confirmation experiments.
v. The minimum values of R_DE_ and A_DE_ achieved by the novel EDM set up for the optimal setting of process parameters were respectively 4.4 and 6.3 times smaller than the corresponding average values: 0.045 mm vs. 0.244 mm for R_DE_ and 0.034 mm vs. 0.247 mm for A_DE_.
vi. The classical EDM set up using a conventional dielectric liquid such as kerosene achieved a poor geometric accuracy during cutting of Ti-6Al-4V through EDM. In particular, mean values of R_DE_ and A_DE_ achieved by the conventional EDM set up were, respectively, 10.2% and 19.4% larger than those obtained by the graphene-mixed dielectric EDM set up. Hence, the blending of graphene particles in the dielectric of EDM has been proven as a good choice for achieving high dimensional accuracy in the machining of Ti-6Al-4V workpieces.

## Figures and Tables

**Table 1:** Chemical composition of the Ti-6Al-4V workpiece.

Elements	Al	V	C	N	O	H	Fe	Y	Other	Ti
wt %	6.75	4.50	0.08	0.05	0.20	0.0125	0.30	0.005	0.40	Balance

**Table 2:** Nominal properties of Ti-6Al-4V workpiece (taken from [15]).

Characteristics	Values
Density (kg/m^3^)	4428.785
Melting Temperature (K)	1882.59–1933.15
Tensile strength (MPa)	869–924
Ultimate tensile strength (MPa)	832
Hardness (HRC)	28–32
Electrical resistivity (Ω/m)	1.724 × 10^−^^6^
Thermal conductivity (Wm/K)	6.7

**Table 3:** Input parameters and their levels.

Input Parameters	Units	1st Level	2nd Level	3rd Level
Polarity	-	Positive	Negative	-
Electrode type	-	Al	Brass	Cu
Spark Voltage	Volt	3	4	5
Discharge Current	Ampere	6	8	10
Pulse time ratio	-	0.5	1.0	1.5
Flushing time	µs	4	6	8

**Table 4:** Relevant properties of graphene nano-particles.

Properties	Units	Magnitude
Density	g/mL	(6–9) × 10^−^^2^
Thickness	nm	2–10
Diameter	μm	2–10
Colour	-	Grey/black powder
Carbon content	%	>99
Electrical conductivity	S/m	80,000
Surface area	m^2^/g	20–40
Additional impurities	wt %	<1
Percentage of water	wt %	<2

**Table 5:** Desired settings for R_DE_ and A_DE_.

Exp. No.	Grey Relational Generation			Grey Relational Coefficients Calculation						GRA Grades Calculation and Alternates’ Ranking	
	R_DE_	A_DE_	(δ)	R_DE_	A_DE_	(δ)	R_DE_ (GC)	A_DE_ (GC)	(δ) (GC)	GRA Grades	GRA Ranking
Xo	1	1	1								
1	0.294	0.173	0.589	0.706	0.827	0.411	0.414	0.377	0.549	0.447	15
2	0.000	0.072	0.795	1.000	0.928	0.205	0.333	0.350	0.709	0.464	12
3	0.158	0.069	0.696	0.842	0.931	0.304	0.372	0.349	0.622	0.448	14
4	0.857	0.859	0.955	0.143	0.141	0.045	0.777	0.780	0.918	0.825	2
5	0.697	0.813	0.536	0.303	0.187	0.464	0.623	0.728	0.519	0.623	6
6	0.578	0.333	0.143	0.422	0.667	0.857	0.542	0.428	0.368	0.446	16
7	0.986	0.857	0.643	0.014	0.143	0.357	0.972	0.777	0.583	0.778	4
8	0.549	0.467	0.768	0.451	0.533	0.232	0.526	0.484	0.683	0.564	10
9	0.611	0.684	0.714	0.389	0.316	0.286	0.562	0.612	0.636	0.604	7
10	1.000	1.000	0.946	0.000	0.000	0.054	1.000	1.000	0.903	0.968	1
11	0.902	0.734	0.482	0.098	0.266	0.518	0.836	0.653	0.491	0.660	5
12	0.413	0.365	0.884	0.587	0.635	0.116	0.460	0.440	0.812	0.571	9
13	0.792	0.785	1.000	0.208	0.215	0.000	0.707	0.700	1.000	0.802	3
14	0.222	0.337	0.598	0.778	0.663	0.402	0.391	0.430	0.554	0.459	13
15	0.119	0.000	0.571	0.881	1.000	0.429	0.362	0.333	0.538	0.411	17
16	0.520	0.651	0.500	0.480	0.349	0.500	0.510	0.589	0.500	0.533	11
17	0.654	0.550	0.696	0.346	0.450	0.304	0.591	0.526	0.622	0.580	8
18	0.122	0.395	0.000	0.878	0.605	1.000	0.363	0.452	0.333	0.383	18

**Table 6:** Optimal parameter settings of graphene-based dielectric EDM set up yielding the lowest values of R_DE_ and A_DE_.

Sr. No.	Control Variables	Graphene Based Dielectric
		Optimal Value
1	Polarity	Negative (−)
2	Electrode material	Al
3	Spark voltage	3V
4	Discharge current	6 A
5	Pulse time ratio	0.5
6	Flushing time	4 µs

**Table 7:** Confirmatory experimental results obtained by implementing optimized EDM’s input parameters.

Responses Magnitude	Radial Dimension Error (R_DE_)	Axial Dimension Error (A_DE_)	Error Difference (δ)
Optimized EDM parameters	0.045 mm	0.034 mm	0.01
Average responses’ value	0.244 mm	0.247 mm	0.04
Improvement	4.4 times	6.3 times	4 times
